# Association of meat consumption with NAFLD risk and liver-related biochemical indexes in older Chinese: a cross-sectional study

**DOI:** 10.1186/s12876-021-01688-7

**Published:** 2021-05-17

**Authors:** Hewei Peng, Xiaoxu Xie, Xinting Pan, Jing Zheng, Yidan Zeng, Xiaoling Cai, Zhijian Hu, Xian-E Peng

**Affiliations:** 1 Department of Epidemiology and Health Statistics, Fujian Provincial Key Laboratory of Environment Factors and Cancer, School of Public Health, Fujian Medical University, Fuzhou, People’s Republic of China; 2 Key Laboratory of Ministry of Education for Gastrointestinal Cancer, Fujian Medical University, Fuzhou, People’s Republic of China

**Keywords:** Non-alcoholic fatty liver disease, Meat consumption, Liver-related biochemical indexes

## Abstract

**Background:**

Non-alcohol fatty liver disease (NAFLD) is the most common liver disease and an unhealthy lifestyle can lead to an increased risk of NAFLD. The present study aims to evaluate the association of meat consumption with NAFLD risk and liver-related biochemical indexes in middle-aged and elderly Chinese.

**Methods:**

A cross-sectional study was conducted in individuals who were 45 years or older and underwent a physical examination from April 2015 to August 2017 in Southeast China. To evaluate associations between meat intake and NAFLD risk, inverse probability of treatment weighting and subgroup analyses were performed with logistic regressions. Spearman’s rank correlation was carried out to examine the relationship between meat consumptions and liver-related biochemical indexes.

**Results:**

High consumptions of red meat (28.44–49.74 and > 71.00 g/day) (*OR*_adjusted_ = 1.948; *P* < 0.001; *OR*_adjusted_ = 1.714; *P* = 0.002) was positively associated with NAFLD risk on inverse probability of treatment weighting analysis, adjusting for smoking, tea intake, weekly hours of physical activity and presence of hypertension, dyslipidemia and diabetes. Exposure–response relationship analysis presented that red meat intake was positively associated with NAFLD risk. Significant associations of red meat intakes with serum levels of γ-glutamyl transferase, alanine transaminase, aspartate aminotransferase, total triglyceride and high-density lipoprotein cholesterol were found (*r*_s_ = 0.176; *P* < 0.001; *r*_s_ = 0.128; *P* < 0.001; *r*_s_ = 0.060; *P* = 0.016; *r*_s_ = 0.085; *P* = 0.001; *r*_s_ = − 0.074; *P* = 0.003).

**Conclusions:**

These findings suggest that the reduction of meat consumption may decrease NAFLD risk and should warrant further investigations.

**Supplementary Information:**

The online version contains supplementary material available at 10.1186/s12876-021-01688-7.

## Background

Non-alcoholic fatty liver disease (NAFLD) is the most common liver disease associated with the metabolic syndrome or its components and is becoming a major global health and economic burden, with a 25% prevalence worldwide [1]. It is defined as the presence of more than 5% of hepatic steatosis, with little or no secondary causes of fatty liver, such as alcohol, virus, and drugs. Besides, NAFLD is associated not only with adverse hepatic outcomes including cirrhosis and liver cancer, but also with non-liver-associated adverse outcomes, such as cardiovascular diseases and diabetes [2–4]. Elderly individuals are the fastest-growing age group worldwide due to great improvements in medications and medical treatments, as well as quality of life. Aging promotes the development of hepatocellular injury and inflammation [5], and the prevalence of NAFLD increases with age [1]. These data highlight a serious concern for the future, and the enormous increasing health burden of NAFLD.

Lifestyle including dietary habits positively influenced the development and progression of NAFLD [6, 7]. Unhealthy dietary patterns including high intakes of soft drinks and meat have been demonstrated to be significantly increased the NAFLD risk [8–11]. Meat is an important source of energy and some indispensable nutrients, including proteins and some micro-nutrients such as iron, zinc, selenium and B-vitamins [12, 13]. However, increased consumption of meat contributes to high intakes of dietary cholesterol, saturated fat, and other harmful compounds, which are closely connected to the NAFLD [14]. The global average per capita consumption of meat is rising, especially in China [12]. Chinese meat consumption increased from 58.9 g/d to 89.7 g/d from 1992 to 2012, which was far beyond the recommendation for Chinese adults [14]. Moreover, high consumption of meat was related to insulin resistance, type 2 diabetes, lipodystrophy, cardiovascular diseases, hepatocellular carcinoma and hypertension[15–18], which coexist and share similar pathogenesis of NAFLD [19, 20]. Therefore, it is important to examine the relationship of meat intake with NAFLD among the Chinese population, especially in middle-aged and elders, who are predisposed to NAFLD [21, 22].

Furthermore, liver-related biochemical parameters such as γ-glutamyl transferase (GGT), aspartate aminotransferase (AST), and alanine transaminase (ALT) were associated with NAFLD [23]. TG/HDL-C was found to be independently associated with fatty liver diseases [24, 25]. However, epidemiological studies regarding the associations of meat subtypes intake with NAFLD risk and liver-related biochemical indexes were not fully addressed.

This cross-sectional study, therefore, is intended to evaluate the associations of meat subtypes intake with the risk of NAFLD and liver-related biochemical indexes among the middle-aged and elder Chinese population.

## Methods

### Study subjects and design

**Fig. 1:**
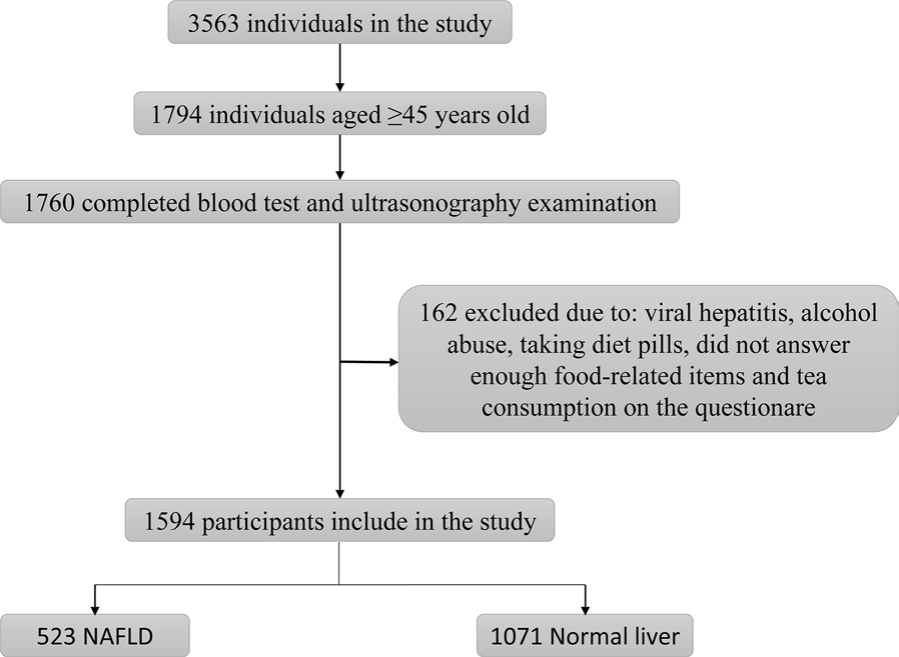
Flowchart of the study population. NAFLD, non-alcoholic fatty liver disease

This was a cross-sectional study, conducted in the health examination center of the Affiliated Nanping First Hospital, Fujian Medical University from April 2015 through August 2017. As shown in Fig. 1, we included individuals aged ≥ 45 years old and who permanently resided in Nanping. Further exclusion criteria in our study were as follows: (A) Individuals whose daily consumptions of alcohol > 40 g (men) or > 20 g (women). (B) Individuals who had any other liver disease history, such as drug-induced liver disease, viral hepatitis, Wilson’s disease, autoimmune hepatitis and total parenteral nutrition. (C) Individuals who were taking hypolipidemic or weight reduction drugs. (D) Individuals who did not answer more than 25 food-related items on the questionnaire. (E) Individuals who did not provide information on smoking and tea consumption. All subjects provided their written informed consent before participating in this study.

The current study was carried out in compliance with the Declaration of Helsinki, and the Ethics Committee of Fujian Medical University approved the study protocol (ethics number 2014096).

### Data collection

#### NAFLD ascertainment

NAFLD was diagnosed by abdominal ultrasonography using established criteria [26]. An abdominal ultrasonography examination was done by experienced radiologists who were unaware of the laboratory and clinical data.

#### Meat intakes assessment

Dietary information on typical food consumption of participants was collected using a semi-quantitative food frequency questionnaire consisting of 110 food-related items, which was developed and validated in a sample from southern China [27]. And the information was obtained from participants interviewed face to face by trained investigators. For each food item, participants used the following response options to indicate how often they ate the selected food on average: 3 times/day, twice/day, once/day, 5–6 times/week, 3–4 times/week, 1–2 times/week, 1–3 times/month, < once/month and rarely. Red meat consisted of pork, beef and lamb. Poultry was composed of chicken and duck. Processed meat contained fried and smoked meat. Fish included: fish, shellfish and crab. The nutritional components of each food item were taken from the *China Food Composition* [28].

#### Covariate assessment

The following variables were self-reported: age, sex, marital status, income, educational level, smoking status, tea intake status, physical activity, medication use and medical conditions. All subjects underwent physical examinations (height, weight, waistline, hipline and blood pressure) and blood tests (fasting plasma-glucose, low-density lipoprotein cholesterol, total cholesterol, TG, HDL-C, AST, ALT and GGT) performed by trained physicians. BMI was calculated as weight/ (height) ^2^. Fatty liver index (FLI), a simple and accurate predictor of hepatic steatosis in the general population, was calculated according to the previous studies: FLI = e ^[0.953^^×^^ln^ ^(TG)^^+^^0.139^^×^^BMI^^+^^0.718^^×^^ln^ ^(GGT)^^+^^0.053^^×^^WC^^−^^15.745]^/ (1 + e ^[0.953^^×^^ln^ ^(TG)^^+^^0.139^^×^^BMI^^+^^0.718^^×^^ln^ ^(GGT)^^+^^0.053^^×^^WC^^−^^15.745]^) × 100. The FLI score range is 0–100, values < 30 rule out and values ≥ 60 rule in steatosis [29]. Participants with a systolic blood pressure ≥ 140 mmHg or diastolic blood pressure ≥ 90 mmHg were defined as having hypertension. And subjects who had one or more of the following abnormalities were diagnosed as dyslipidemia: total cholesterol ≥ 6.2 mmol/L, TG > 2.25 mmol/L, low-density lipoprotein cholesterol > 4.13 mmol/L or HDL-C < 1.03 mmol/L [30]. Diabetes was diagnosed as follows: fasting plasma glucose of 7.0 mmol/L or greater or 2-h postprandial glucose greater than or equal to 11.1 mmol/L.

### Statistical analysis

Participants were classified into 4 groups based on the quartile of total meat consumption. The baseline characteristics of subjects were analyzed using the Nonparametric Kruskal–Wallis test for non-normal continuous variables and Chi-Square test for nominal variables. Continuous variables were expressed as median (interquartile range, *IQR*). Propensity scores were used to explain potential confounders by observed characteristics at baseline [31]. Age, gender and BMI were used to calculate the propensity score. Inverse probability of treatment weighting analysis was performed to evaluate associations of red meat, processed meat, poultry and fish intakes with NAFLD, adjusting for smoking status, tea intake status, weekly hours of physical activity, and presence of hypertension, dyslipidemia and diabetes. The lowest quartile (*Q*_1_) of each type of meat intake was served as the reference group and *P* for trend was calculated by setting the meat intake quartiles as a continuous variable. To evaluate dose–response relationships between continuous exposure variables (red meat, processed meat, poultry and fish intakes) and NAFLD, a logistic model with restricted cubic spline using five knots at 0.05, 0.275, 0.5, 0.725 and 0.95 was built, adjusting for age, sex, BMI, smoking status, tea intake status, weekly hours of physical activity, and presence of hypertension, dyslipidemia and diabetes.

We also performed subgroup analysis to examine relationships of red meat, processed meat, poultry, and fish with NAFLD by the following subgroups: age (< 60 years or ≥ 60 years), gender (men or women), BMI (< 24 kg/m^2^ or ≥ 24 kg/m^2^), smoking status (never, former or current), tea consumption status (yes or no), hypertension (yes or no), dyslipidemia (yes or no), diabetes (yes or no), and weekly hours of physical activity (< 9 h/week or ≥ 9 h/week). *P* value for interaction was calculated. Two sensitivity analyses were conducted: (1) logistic regression analysis without IPTW; and (2) propensity score-matching logistic regressions. Furthermore, to investigate the associations of meat subtypes intakes with the concentrations of serum GGT, ALT, AST, fasting plasma glucose, total cholesterol, TG, low-density lipoprotein cholesterol and HDL-C, spearman’s rank correlation was performed.

For statistical analyses, SPSS, version 19.0.0.1(IBM SPSS, 2010, Chicago, IL, USA) and R, version 4.0.0 (R Project for Statistical Computing) were performed. All *P* values were two-tailed and results were considered to be statistically significant at *P* values < 0.05.

## Results

### Baseline characteristics

A total of 1594 individuals were included in this analysis. In the entire study sample, 53.5% were men, the average age was 53.54 ± 6.90 years and the average body mass index (BMI) was 23.77 ± 2.99 kg/m^2^. As shown in Table 1, compared with participants with lower total intakes of red, processed meat, poultry and fish, participants with higher total intakes (1) were younger and more likely to be married persons, male, smokers, and tea drinkers; (2) had higher educational level, higher income, higher BMI, higher waist and hip circumference; (3) had higher levels of plasma γ-glutamyl transferase and alanine aminotransferase; and (4) had a higher prevalence rate of NAFLD (each *P* < 0.05).

**Table 1:** Baseline characteristics of the study population by quartiles of the total intake of red, processed meat, poultry and fish

	*Q*_1_ (< 65.12 g/day)	*Q*_2_ (65.12–95.00 g/day)	*Q*_3_ (95.01–150.80 g/day)	*Q*_4_ (> 150.80 g/day)	*P*
Age (years), *M* (*IQR*)	53 (49–59)	52 (49–58)	52 (48–57)	52 (48–57)	0.010
Gender, n (%)					< 0.001
Male	129 (39.7)	223 (47.2)	237 (59.4)	263 (66.1)	
Female	196 (60.3)	249 (52.8)	162 (40.6)	135 (33.9)	
NAFLD, n (%)	84 (25.8)	151 (32.0)	136 (34.1)	152 (38.2)	0.005
Educational level, n (%)					< 0.001
Primary school and less than	71 (21.8)	71 (15.0)	40 (10.00)	34 (8.5)	
Junior middle and high school	154 (47.4)	228 (48.3)	197 (49.4)	209 (52.5)	
Junior college or above	100 (30.8)	173 (36.7)	162 (40.6)	155 (38.9)	
Income (yuan/month), n (%)					< 0.001
< 2000	52 (16.0)	36 (7.6)	28 (7.0)	24 (6.0)	
2000–3000	131 (40.3)	167 (35.4)	121 (30.3)	120 (30.2)	
> 3000	142 (43.7)	269 (57.0)	250 (62.7)	254 (63.8)	
Marital status, n (%)					< 0.001
Married	312 (96.0)	469 (99.4)	391 (98.0)	397 (99.7)	
Single or other	13 (4.0)	3 (0.6)	8 (2.0)	1 (0.3)	
Smoking status, n (%)					< 0.001
Never	264 (81.2)	382 (80.9)	283 (70.9)	248 (62.3)	
Former	17 (5.2)	20 (4.2)	24 (6.0)	34 (8.55)	
Current	44 (13.5)	70 (14.8)	92 (23.11)	116 (29.1)	
Tea intake status, n (%)					< 0.001
No	168 (51.7)	218 (46.2)	147 (36.8)	136 (34.2)	
Yes	157 (48.3)	254 (53.8)	252 (63.2)	262 (65.8)	
BMI (kg/m^2^), *M* (*IQR*)	22.72 (21.06–24.98)	23.60 (21.64–25.40)	23.41 (21.48–25.64)	24.10 (22.49–26.09)	< 0.001
Waist circumference (cm), *M* (*IQR*)	83 (76–89)	83 (77–90)	85 (78–91)	86 (80–92)	< 0.001
Hip circumference (cm), *M* (*IQR*)	94 (91–98)	95 (91–99)	96 (92–99)	97 (93–101)	< 0.001
SBP (mmHg), *M* (*IQR*)	124 (112–140)	120 (110–132)	120 (110–131)	122 (112–134)	0.124
DBP (mmHg), *M* (*IQR*)	80 (74–88)	80 (77–88)	80 (75–88)	80 (76–90)	0.317
Hypertension, n (%)	132 (40.6)	170 (36.0)	148 (37.1)	157 (39.4)	0.528
Dyslipidemia, n (%)	121 (37.2)	165 (35.0)	144 (36.1)	162 (40.7)	0.345
Diabetes, n (%)	66 (20.3)	96 (20.3)	77 (19.3)	74 (18.6)	0.909
GGT (U/L), *M* (*IQR*)	22 (17–33)	24 (17–36)	26 (18–39)	28 (20–44)	< 0.001
ALT (U/L), *M* (*IQR*)	19 (14–25)	20 (15–27)	21 (16–29)	21 (16–30)	0.001
AST (U/L), *M* (*IQR*)	22 (19–26)	22 (19–26)	22 (19–26)	23 (19–27)	0.318
FPG (mg/dL), *M* (*IQR*)	469.44 (441.98–513.73)	467.67 (443.76–516.39)	467.67 (438.44–505.76)	469.44 (441.10–510.19)	0.708
TC (mg/dL), *M* (*IQR*)	460.58 (410.98–505.76)	467.67 (416.30–513.73)	465.90 (420.73–528.79)	460.58 (420.73–523.47)	0.328
TG ( mg/dL), *M* (*IQR*)	119.57 (85.92–170.95)	127.55 (91.23–174.49)	126.66 (92.12–178.03)	130.20 (93.00–183.35)	0.354
LDL (mg/dL), *M* (*IQR*)	293.18 (239.15–339.24)	296.72 (248.89–344.55)	293.18 (248.89–350.75)	294.07 (239.15–336.58)	0.610
HDL (mg/dL), *M* (*IQR*)	116.92 (101.86–129.32)	116.92 (100.97–129.32)	116.92 (100.97–129.32)	114.26 (98.32–127.55)	0.124
Physical activity (hours/week), *M* (*IQR*)	10.5 (4.5–17.5)	9.5 (4.0–15.3)	8.9 (4.1–15.0)	7.0 (3.5–15.6)	0.051

BMI, body mass index; SBP, systolic blood pressure; DBP, diastolic blood pressure; GGT, γ-glutamyl transferase; ALT, alanine aminotransferase; AST, aspartate aminotransferase; FPG, fasting plasma-glucose; TC, total cholesterol; TG, total triglyceride; LDL, low-density lipoprotein; HDL, high-density lipoprotein; NAFLD, non-alcoholic fatty liver disease; *M* (*IQR*) = Median (Interquartile range); *Q* = quartile

### Meat subtypes consumptions and NAFLD

As presented in Table 2, after propensity score weighting, red meat intake was positively related to the risk of NAFLD (*OR* per 50 g/day of red meat: 1.128, 95% *CI: *1.005–1.266). After further adjustment for smoking status, tea intake status, weekly hours of physical activity and the presence of hypertension, dyslipidemia, and diabetes, the association with NAFLD remained significant (*OR*_adjusted_ = 1.143). High consumptions of red (*Q*_2_ and *Q*_4_) were significantly relevant to higher odds for NAFLD (*OR*_adjusted_ = 1.948 and 1.716, respectively), adjusting for those potential confounders. Furthermore, the results did not change appreciably after further adjusted for energy and cholesterol intakes (see Additional file 1: Table S1).

**Table 2:** Propensity score weighted univariable and multivariable analysis of associations between meat subtypes consumptions and NAFLD

Variable	Crude *OR* (95%*CI*)	Adjusted *OR* (95%*CI*)^a^
*Red meat*		
Continuous variable		
Per 50 g/day increase	1.128 (1.005–1.266)	1.143 (1.010–1.294)
Categorical variables		
*Q*_1_(< 28.44 g/day)	1 (Reference)	1 (Reference)
*Q*_2_(28.44–49.74 g/day)	1.901 (1.386–2.609)	1.948 (1.399–2.714)
*Q*_3_ (49.75–71.00 g/day)	1.221 (0.872–1.710)	1.190 (0.833–1.698)
*Q*_4_ (> 71.00 g/day)	1.659 (1.199–2.295)	1.716 (1.214–2.424)
*P* for trend	0.043	0.043
*Processed meat*		
Continuous variable		
Per 50 g/day increase	1.003 (0.806–1.247)	0.965 (0.766–1.216)
Categorical variables		
*Q*_1_ (< 2.26 g/day)	1 (Reference)	1 (Reference)
*Q*_2_ (2.26–4.61 g/day)	1.159 (0.828–1.622)	1.146 (0.806–1.629)
*Q*_3_ (4.62–6.59 g/day)	1.521 (1.104–2.096)	1.389 (0.992–1.946)
*Q*_4_ (> 6.59 g/day)	1.376 (0.986–1.921)	1.335 (0.938–1.901)
*P* for trend	0.020	0.059
*Poultry*		
Continuous variable		
Per 50 g/day increase	0.974 (0.666–1.424)	1.021 (0.681–1.531)
Categorical variables		
*Q*_1_(< 7.00 g/day)	1 (Reference)	1 (Reference)
*Q*_2_ (7.00–9.00 g/day)	1.145 (0.829–1.582)	1.131 (0.805–1.589)
*Q*_3_ (9.01–14.00 g/day)	1.086 (0.806–1.462)	0.998 (0.730–1.364)
*Q*_4_ (> 14.00 g/day)	1.052 (0.774–1.431)	1.078 (0.779–1.493)
*P* for trend	0.753	0.801
*Fish*		
Continuous variable		
Per 50 g/day increase	0.967 (0.868–1.077)	0.989 (0.883–1.108)
Categorical variables		
*Q*_1_ (< 11.01 g/day)	1 (Reference)	1 (Reference)
*Q*_2_ (11.01–25.15 g/day)	0.865 (0.632–1.184)	0.870 (0.625–1.211)
*Q*_3_ (25.16–50.42 g/day)	1.007 (0.735–1.379)	1.013 (0.727–1.411)
*Q*_4_ (> 50.42 g/day)	0.922 (0.669–1.271)	0.943 (0.671–1.324)
*P* for trend	0.870	0.976

NAFLD, non-alcoholic fatty liver diseases

^a^Adjusted by smoking status, tea intake status, weekly hours of physical activity and the presence of hypertension, dyslipidemia and diabetes. *Q* = quartile

**Fig. 2:**
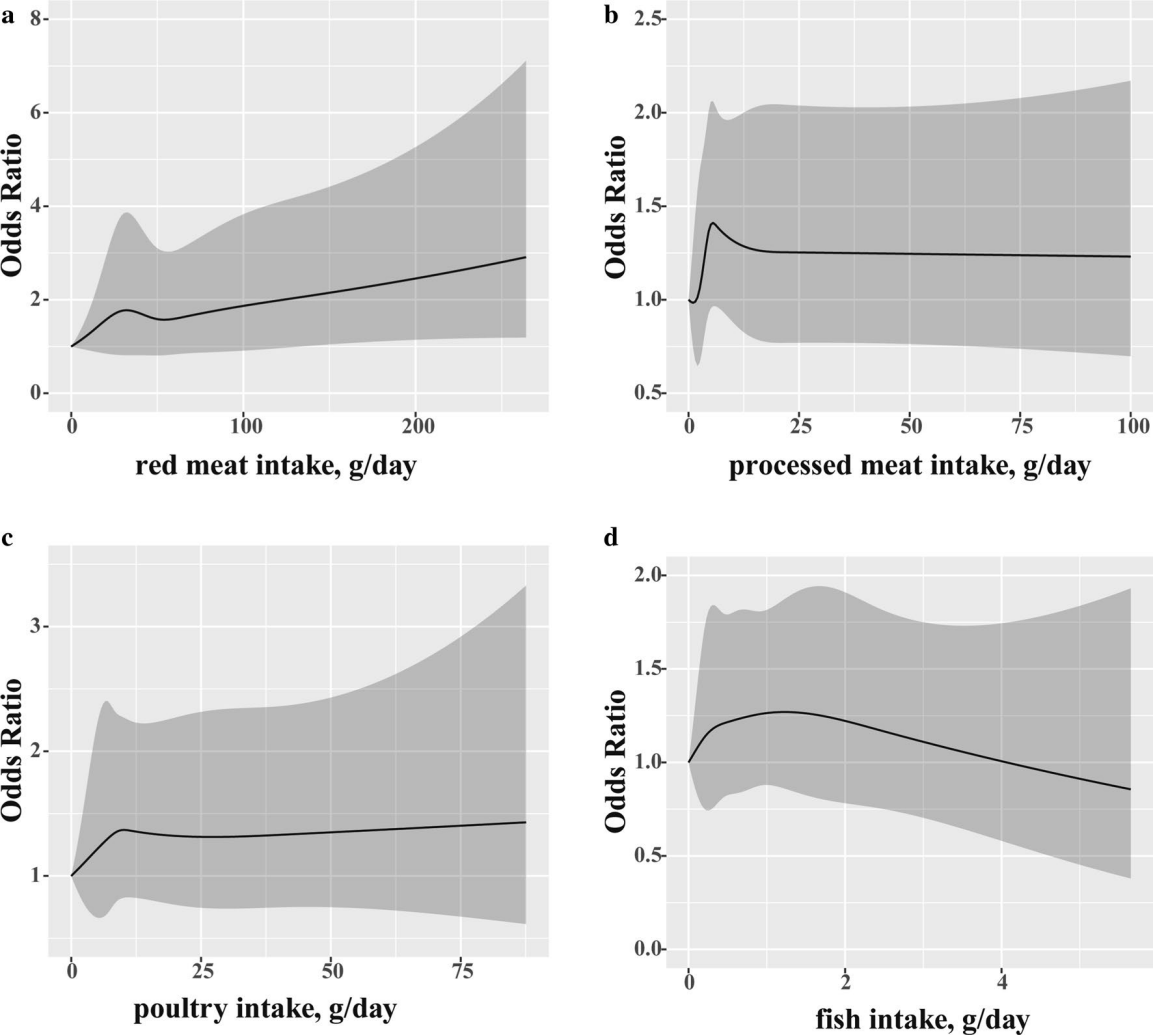
Restricted cubic spline model of the odds ratios of non-alcoholic fatty liver diseases (NAFLD) with intakes of (**a**) red meat (*P*_nonlinearity_ = 0.63), (**b**) processed meat (*P*_nonlinearity_ = 0.24), (**c**) poultry (*P*_nonlinearity_ = 0.69) and (**d**) fish (*P*_nonlinearity_ = 0.55). Gray area, 95% confidence interval. Each model was adjusted for age, gender, body mass index (BMI), smoking status, tea intake status, weekly hours of physical activity and the presence of hypertension, dyslipidemia and diabetes

The restricted cubic splines analysis was applied to explain the exposure–response association between meat subtypes intakes and the risk of NAFLD (Fig. 2). The *OR*s of NAFLD increased with red meat and poultry consumptions but decreased with the fish intake (Fig. 2a, c, d). Moreover, the trend for *OR* of processed meat intake was not found (Fig. 2b).

### Stratified analyses

The positive association between meat intake and NAFLD was consistent across strata of age, sex, smoking status, tea intake status, BMI, weekly hours of physical activity, and the presence of hypertension, dyslipidemia and diabetes. Significant associations between red meat intake and the risk of NAFLD were not only found in males but also existed in people whose BMI ≥ 24 kg/m^2^, tea-drinkers, people with hypertension and people without dyslipidemia. Adjusted *OR*s were estimated to be 1.177, 1.195, 1.229, 1.304 and 1.203, respectively. Moreover, fish intake was found positively relevant to NAFLD in people with diabetes (*OR*_adjusted_ = 1.447). Nonetheless, no significant interactions of meat subtypes intakes and potential confounders were identified (Table 3).

**Table 3:** Stratified analysis of association between meat subtypes intakes and NAFLD risk

Subgroup	Red meat (per 50 g/day)		Processed meat (per 50 g/day)		Poultry (per 50 g/day)		Fish (per 50 g/day)	
	*OR* (95%*CI*)	*P* for interaction	*OR* (95%*CI*)	*P* for interaction	*OR* (95%*CI*)	*P* for interaction	*OR* (95%*CI*)	*P* for interaction
Gender		0.259		0.759		0.565		0.844
Male	1.177 (1.013–1.368)		0.991 (0.798–1.231)		1.156 (0.737–1.838)		0.962 (0.820–1.128)	
Female	1.097 (0.883–1.364)		1.165 (0.619–2.193)		0.896 (0.397–2.018)		1.009 (0.857–1.188)	
Age		0.503		0.208		0.121		0.220
< 60 years	1.112 (0.969–1.276)		1.004 (0.812–1.243)		0.990 (0.649–1.510)		0.956 (0.841–1.087)	
≥ 60 years	1.255 (0.956–1.649)		3.397 (0.657–17.567)		2.134 (0.686–6.636)		1.201 (0.870–1.657)	
BMI		0.543		0.346		0.391		0.947
< 24 kg/m^2^	1.141 (0.906–1.438)		0.856 (0.508–1.441)		1.124 (0.624–2.024)		1.025 (0.873–1.205)	
≥ 24 kg/m^2^	1.195 (1.029–1.387)		1.196 (0.789–1.811)		1.542 (0.880–2.704)		1.002 (0.855–1.175)	
Smoking status		0.425		0.811		0.746		0.732
Never	1.079 (0.916–1.271)		1.195 (0.685–2.082)		1.332 (0.747–2.376)		1.018 (0.888–1.168)	
Former	1.560 (0.908–2.680)		1.422 (0.052–39.060)		1.246 (0.228–6.818)		1.047 (0.697–1.573)	
Current	1.176 (0.959–1.442)		1.006 (0.798–1.269)		0.936 (0.535–1.638)		0.919 (0.726–1.164)	
Tea intake status		0.095		0.842		0.201		0.745
No	1.014 (0.832–1.235)		1.006 (0.808–1.253)		0.822 (0.430–1.570)		0.965 (0.726–1.281)	
Yes	1.229 (1.048–1.442)		1.037 (0.499–2.155)		1.413 (0.838–2.385)		0.994 (0.875–1.131)	
Hypertension		0.166		0.195		0.363		0.094
No	1.037 (0.884–1.216)		1.269 (0.870–1.852)		0.935 (0.568–1.537)		0.905 (0.760–1.079)	
Yes	1.304 (1.063–1.598)		0.766 (0.389–1.508)		1.440 (0.752–2.757)		1.138 (0.945–1.369)	
Dyslipidemia		0.245		0.118		0.607		0.709
No	1.203 (1.017–1.424)		1.686 (0.806–3.526)		1.208 (0.709–2.056)		0.999 (0.860–1.159)	
Yes	1.072 (0.898–1.278)		0.958 (0.759–1.211)		1.037 (0.594–1.812)		0.975 (0.811–1.172)	
Diabetes		0.973		0.852		0.112		0.110
No	1.129 (0.988–1.290)		1.001 (0.810–1.237)		0.978 (0.631–1.518)		0.936 (0.813–1.076)	
Yes	1.272 (0.923–1.753)		1.206 (0.357–4.073)		2.816 (0.990–8.008)		1.447 (1.017–2.059)	
Physical activity		0.745		0.730		0.653		0.736
< 9.0 h/week	1.135 (0.949–1.356)		0.800 (0.346–1.851)		1.211 (0.716–2.046)		1.001 (0.828–1.211)	
≥ 9.0 h/week	1.134 (0.957–1.344)		1.024 (0.829–1.266)		1.070 (0.600–1.908)		0.986 (0.851–1.142)	

NAFLD, non-alcoholic fatty liver diseases; BMI, body mass index

^*^Adjusted for age, gender, BMI, smoking status, tea intake status, weekly hours of physical activity and the presence of hypertension, dyslipidemia and diabetes

### Sensitivity analyses

Results from unweighted analysis was similar to those from propensity score-weighted analysis. Compared with the lowest quartile of meat intake, high intakes of red meat (*Q*_2_ and *Q*_4_) were significantly associated with NAFLD risk without the inverse probability of treatment weighting (*OR*_adjusted_ = 1.484 and 1.558, respectively) (see Additional file 2: Table S2). Nonetheless, positive associations of red meat (*Q*_4_) and processed meat (*Q*_3_) consumptions with NAFLD risk were observed on propensity score matching analysis, with adjusted *OR* of 1.673 and 1.800, respectively (see Additional file 3: Table S3).

### Meat subtypes intakes and liver-related biochemical indexes

**Fig. 3:**
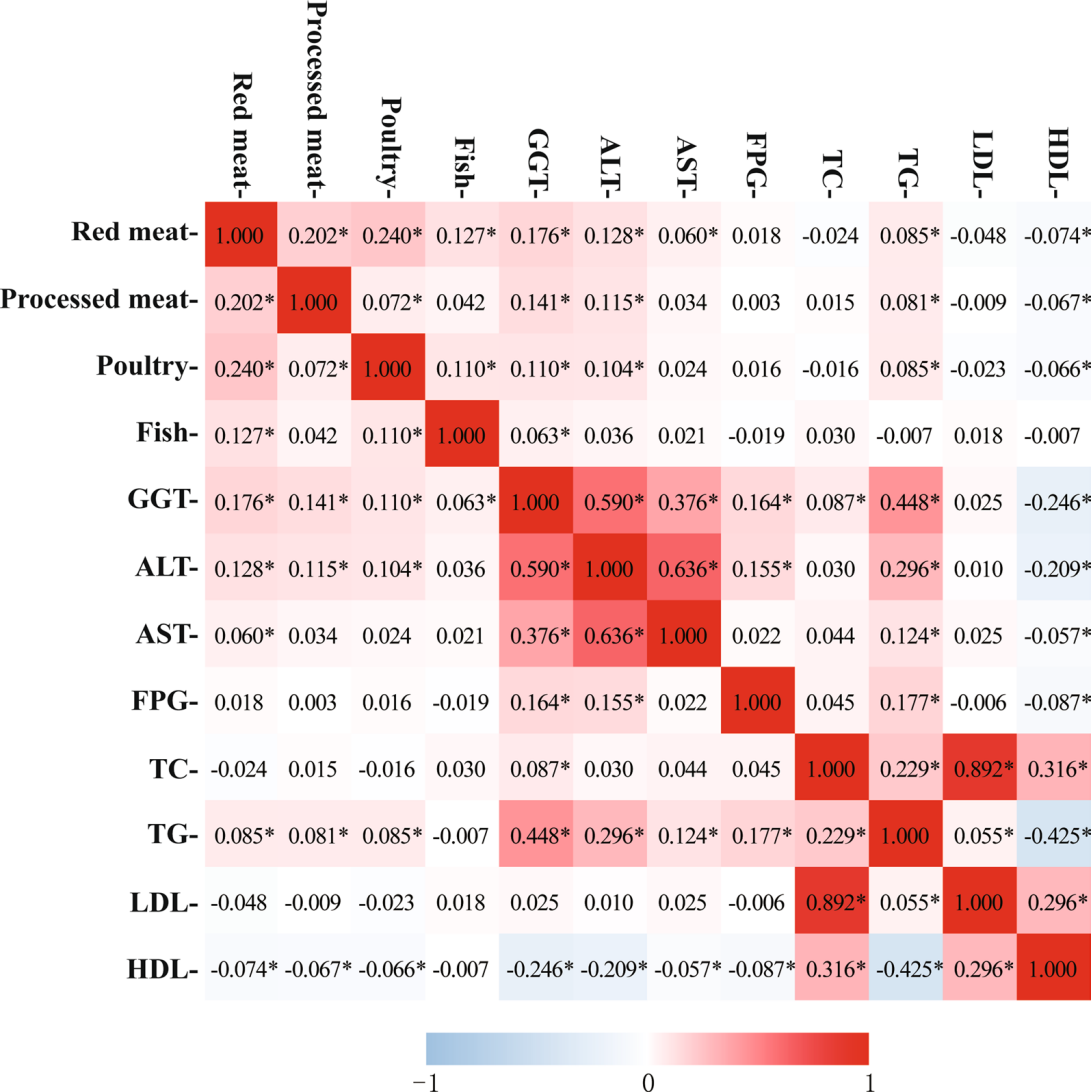
Heatmap of associations between meat subtypes intakes and liver-related biochemical indexes. GGT, γ-glutamyl transferase; ALT, alanine aminotransferase; AST, aspartate aminotransferase; FPG, fasting plasma-glucose; TC, total cholesterol; TG, total triglyceride; LDL, low-density lipoprotein; HDL, high-density lipoprotein. *P < 0.05

Associations between meat subtypes intakes and liver-related biochemical indexes were shown in Fig. 3. Red meat intake was positively correlated with levels of GGT, ALT, AST and total triglyceride (TG), but inversely with high-density lipoprotein cholesterol (HDL-C) (Spearman test correlation coefficient = 0.176, 0.128, 0.060, 0.085 and − 0.074, respectively). Processed meat intake was significantly associated with GGT, ALT, TG and HDL-C (Spearman test correlation coefficient = 0.141, 0.115, 0.081 and − 0.067, respectively). Similarly, significant associations were observed between poultry intake and GGT, ALT, TG and HDL-C (Spearman test correlation coefficient = 0.111, 0.104, 0.085 and − 0.066, respectively). Besides, GGT was also found to be significantly related to the consumption of fish (Spearman test correlation coefficient = 0.063).

## Discussion

In this cross-sectional study, we observed that NAFLD was associated with higher intake levels of red meat. Significant associations of serum levels of GGT, ALT, AST, TG and HDL-C with meat subtypes intakes were found as well. Additionally, no significant interactions between meat consumptions and potential confounders for NAFLD were detected.

Significant associations between high meat consumptions and NAFLD were demonstrated in a few studies [8, 10, 11, 32]. Our results are in accordance with the previous studies, indicating a positive association between high red meat intake and NAFLD. Two cross-sectional studies have presented that red meat was significantly correlated with NAFLD [8, 11]. In another cross-sectional study, high intakes of total meat, especially red meat and/or processed meat were positively linked to NAFLD and insulin resistance, while processed meat alone was only relevant to insulin resistance. This is mainly due to a relatively low level of processed meat consumption in their research set [10]. In addition, a nested case–control study also showed that high consumptions of red meat, processed red meat and poultry were positively associated with NAFLD [32]. Due to the better accuracy of the continuous *OR*s [33], the dose–response analysis we have employed can better measure the overall trends of the *OR*s for meat intakes. The 95% *CI* for red meat intake beyond 200 g/day was slightly wider, because that less than 2% of participants had red meat intake > 200 g/day, that was, the tendency of red meat intake within the range 0–200 g/day was relatively reliable and stable.

There are several plausible mechanisms by which meat intake is related to NAFLD. NAFLD was reported to be closely linked to hepatic insulin resistance, which had a strong correlation with liver-related biochemical indexes such as ALT, AST and GGT [34]. GGT and ALT had been considered as biomarkers of hepatic fat accumulation, which can lead to hepatic insulin resistance and increase the contribution of gluconeogenesis to total endogenous glucose production [35]. A cross-sectional study of 2198 European reported a significant positive association between red meat and GGT. As GGT is also a potential nonspecific marker of oxidative stress, the author suggested that oxidative stress may play a vital role underlying the development of chronic diseases with red meat intake [36]. Another cross-sectional study indicated that TG/HDL-C was independently relevant to the risk of NAFLD. The author attributed this result to insulin resistance [37]. Positive associations of serum levels of GGT, ALT and TG with red meat intakes were found in this study. Inversely, serum HDL-C concentration was negatively relevant to red meat and processed meat intakes. Hence, it’s plausible that increased hepatic lipid accumulation and insulin resistance play a substantial role in the relationship of meat intakes with the development of NAFLD. In addition, a study by Avila et al*.* found that red meat was positively relevant to serum ferritin [38], which can increase the risk of NAFLD [39, 40]. Fried food will produce some hazardous chemicals, such as advanced glycation end products and trans fatty acids [41, 42], which were reported to play a critical role in NAFLD [43, 44].

Significant associations were found in several subgroups. A positive association was observed between red meat and NAFLD in males, perhaps it is because males had higher meat intakes and a higher prevalence of fatty liver than females [45]. Moreover, several studies had found significant associations of high meat intakes with obesity, type 2 diabetes and hypertension [15, 18, 46], which were considered as risk factors in the development of NAFLD [20, 47, 48]. In our study, a positive association of red meat intake with NAFLD was found in people with BMI ≥ 24 kg/m^2^ and people with hypertension.

This study, however, had several limitations. Firstly, because of the cross-sectional study design, the casual inference was not allowed. Secondly, measurement error was unavoidable for self-reported diet and other data. Nevertheless, since all participants and researchers in this study were blinded to the results of abdominal ultrasonography and blood test, a reporting bias without differences is likely only to attenuate our observed association. Thirdly, since the study subjects were middle-aged and elders, it should be cautious in generalizing our findings to the wider population. And population-wide prospective studies were needed. Fourthly, in our study, the evaluation of the presence of NAFLD was performed only by an abdominal ultrasonography examination, which is not sensitive enough to detect mild steatosis. As the absence of the information on the severity of hepatic steatosis, we were unable to investigate the association of NASH with meat consumption. However, ultrasonography examination can provide a non-invasive prediction of liver histology which in moderate and severe steatosis and advanced fibrosis can be both highly sensitive and specific. Moreover, ultrasonography examination was done by the same experienced radiologists who were unaware of the laboratory and clinical data in our study. Hence, this potential non-differential bias can only weaken the observed associations. Besides, we have evaluated the association between meat consumptions and FLI, the results remained the same (see Additional file 4: Table S4). Lastly, although a comprehensive set of confounders were considered, as an observational study, the presence of unmeasured confounders is possible. For example, as thyroid function was reported to be associated with NAFLD risk [49, 50], related data were lack in our study. Although we have excluded participants who were taking hypolipidaemic or weight reduction drugs, as well as individuals who had any other liver disease history, such as drug-induced liver disease, viral hepatitis, Wilson’s disease, autoimmune hepatitis and total parenteral nutrition, the possible interference of other drugs may exist. However, we have performed subgroup analysis to examine the relationships of meat consumption with NAFLD by the following subgroups: age, sex, BMI, smoking status, tea consumption status, hypertension, dyslipidemia, diabetes, and weekly hours of physical activity. Sensitivity analyses were also used to examine the association of meat intakes with NAFLD.

## Conclusions

In conclusion, a positive relationship between high consumptions of red meat and the risk of NAFLD was observed. In addition, serum levels of liver-related biochemical indexes were significantly relevant to red meat intake. Our findings suggested that the reduction of meat consumption may decrease the risk of NAFLD.

## Supplementary Information

**Additional file 1: Table S1.** Propensity score weighted univariable and multivariable analysis of associations between meat subtypes consumptions and NAFLD

**Additional file 2: Table S2.** Univariable and multivariable analysis of associations between meat subtypes consumptions and NAFLD

**Additional file 3: Table S3.** Propensity score-matched univariable and multivariable analysis of associations between meat subtypes consumptions and NAFLD

**Additional file 4: Table S4.** Results of propensity score weighted univariable and multivariable ordinal logistic model using three levels of FLI as response

## Data Availability

The datasets used can be available from the corresponding author on reasonable request.

## Abbreviations

NAFLD: Non-alcoholic fatty liver disease
BMI: Body mass index
GGT: γ-Glutamyl transferase
AST: Aspartate aminotransferase
ALT: Alanine transaminase
TG: Total triglyceride
HDL-C: High-density lipoprotein cholesterol
IQR: Interquartile range
IPTW: Inverse probability of treatment weighting analysis

## References

[1] Younossi ZM, Koenig AB, Abdelatif D, Fazel Y, Henry L, Wymer M (2016). Global epidemiology of nonalcoholic fatty liver disease-meta-analytic assessment of prevalence, incidence, and outcomes. Hepatology.

[2] Friedman SL, Neuschwander-Tetri BA, Rinella M, Sanyal AJ (2018). Mechanisms of NAFLD development and therapeutic strategies. Nat Med.

[3] Anstee QM, Targher G, Day CP (2013). Progression of NAFLD to diabetes mellitus, cardiovascular disease or cirrhosis. Nat Rev Gastroenterol Hepatol.

[4] Gaggini M, Morelli M, Buzzigoli E, DeFronzo RA, Bugianesi E, Gastaldelli A (2013). Non-alcoholic fatty liver disease (NAFLD) and its connection with insulin resistance, dyslipidemia, atherosclerosis and coronary heart disease. Nutrients.

[5] Fontana L, Zhao E, Amir M, Dong H, Tanaka K, Czaja MJ (2013). Aging promotes the development of diet-induced murine steatohepatitis but not steatosis. Hepatology.

[6] Romero-Gómez M, Zelber-Sagi S, Trenell M (2017). Treatment of NAFLD with diet, physical activity and exercise. J Hepatol.

[7] Eslamparast T, Tandon P, Raman M (2017). Dietary composition independent of weight loss in the management of non-alcoholic fatty liver disease. Nutrients.

[8] Zelber-Sagi S, Nitzan-Kaluski D, Goldsmith R, Webb M, Blendis L, Halpern Z, Oren R (2007). Long term nutritional intake and the risk for non-alcoholic fatty liver disease (NAFLD): a population based study. J Hepatol.

[9] Oddy WH, Herbison CE, Jacoby P, Ambrosini GL, O'Sullivan TA, Ayonrinde OT (2013). The Western dietary pattern is prospectively associated with nonalcoholic fatty liver disease in adolescence. Am J Gastroenterol.

[10] Zelber-Sagi S, Ivancovsky-Wajcman D, Fliss Isakov N, Webb M, Orenstein D, Shibolet O, Kariv R (2018). High red and processed meat consumption is associated with non-alcoholic fatty liver disease and insulin resistance. J Hepatol.

[11] Liu X, Peng Y, Chen S, Sun Q (2018). An observational study on the association between major dietary patterns and non-alcoholic fatty liver disease in Chinese adolescents. Medicine (Baltimore).

[12] Godfray HCJ, Aveyard P, Garnett T, Hall JW, Key TJ, Lorimer J (2018). Meat consumption, health, and the environment. Science.

[13] Ekmekcioglu C, Wallner P, Kundi M, Weisz U, Haas W, Hutter H-P (2018). Red meat, diseases, and healthy alternatives: a critical review. Crit Rev Food Sci Nutr.

[14] He Y, Yang X, Xia J, Zhao L, Yang Y (2016). Consumption of meat and dairy products in China: a review. Proc Nutr Soc.

[15] Micha R, Michas G, Mozaffarian D (2012). Unprocessed red and processed meats and risk of coronary artery disease and type 2 diabetes—an updated review of the evidence. Curr Atheroscler Rep.

[16] Zhong VW, van Horn L, Greenland P, Carnethon MR, Ning H, Wilkins JT (2020). Associations of processed meat, unprocessed red meat, poultry, or fish intake with incident cardiovascular disease and all-cause mortality. JAMA Intern Med.

[17] Freedman ND, Cross AJ, McGlynn KA, Abnet CC, Park Y, Hollenbeck AR (2010). Association of meat and fat intake with liver disease and hepatocellular carcinoma in the NIH-AARP cohort. J Natl Cancer Inst.

[18] Schwingshackl L, Schwedhelm C, Hoffmann G, Knüppel S, Iqbal K, Andriolo V (2017). Food groups and risk of hypertension: a systematic review and dose–response meta-analysis of prospective studies. Adv Nutr.

[19] Francque SM, van der Graaff D, Kwanten WJ (2016). Non-alcoholic fatty liver disease and cardiovascular risk: pathophysiological mechanisms and implications. J Hepatol.

[20] Lonardo A, Nascimbeni F, Mantovani A, Targher G (2018). Hypertension, diabetes, atherosclerosis and NASH: cause or consequence?. J Hepatol.

[21] Marchisello S, Di Pino A, Scicali R, Urbano F, Piro S, Purrello F, Rabuazzo AM (2019). Pathophysiological, molecular and therapeutic issues of nonalcoholic fatty liver disease: an overview. Int J Mol Sci.

[22] Frith J, Day CP, Henderson E, Burt AD, Newton JL (2009). Non-alcoholic fatty liver disease in older people. Gerontology.

[23] Ndrepepa G, Colleran R, Kastrati A (2018). Gamma-glutamyl transferase and the risk of atherosclerosis and coronary heart disease. Clin Chim Acta.

[24] Chen Z, Qin H, Qiu S, Chen G, Chen Y (2019). Correlation of triglyceride to high-density lipoprotein cholesterol ratio with nonalcoholic fatty liver disease among the non-obese Chinese population with normal blood lipid levels: a retrospective cohort research. Lipids Health Dis.

[25] Fukuda Y, Hashimoto Y, Hamaguchi M, Fukuda T, Nakamura N, Ohbora A (2016). Triglycerides to high-density lipoprotein cholesterol ratio is an independent predictor of incident fatty liver; a population-based cohort study. Liver Int.

[26] de Zeng M, Fan JG, Lu LG, Li YM, Chen CW, Wang BY, Mao YM (2008). Guidelines for the diagnosis and treatment of nonalcoholic fatty liver diseases. J Dig Dis.

[27] Ke L, Toshiro T, Fengyan S, Ping Y, Xiaoling D, Kazuo T (2005). Relative validity of a semi-quantitative food frequency questionnaire versus 3 day weighed diet records in middle-aged inhabitants in Chaoshan area, China. Asian Pac J Cancer Prev.

[28] Yuexin Y, Peking NFS, Guangya W, Xingchang P. China food composition 2002. 2002.

[29] Bedogni G, Bellentani S, Miglioli L, Masutti F, Passalacqua M, Castiglione A, Tiribelli C (2006). The Fatty Liver Index: a simple and accurate predictor of hepatic steatosis in the general population. BMC Gastroenterol.

[30] Kopin L, Lowenstein C (2017). Dyslipidemia. Ann Intern Med.

[31] Grool AM, Aglipay M, Momoli F, Meehan WP, Freedman SB, Yeates KO (2016). Association between early participation in physical activity following acute concussion and persistent postconcussive symptoms in children and adolescents. JAMA.

[32] Noureddin M, Zelber-Sagi S, Wilkens LR, Porcel J, Boushey CJ, Le Marchand L (2019). Diet associations with nonalcoholic fatty liver disease in an ethnically diverse population: the multiethnic cohort. Hepatology.

[33] Yu J, Tao Y, Dou J, Ye J, Yu Y, Jin L (2018). The dose–response analysis between BMI and common chronic diseases in northeast China. Sci Rep.

[34] Sesti G, Fiorentino TV, Hribal ML, Sciacqua A, Perticone F (2013). Association of hepatic insulin resistance indexes to nonalcoholic fatty liver disease and related biomarkers. Nutr Metab Cardiovasc Dis.

[35] Samuel VT, Liu Z-X, Qu X, Elder BD, Bilz S, Befroy D (2004). Mechanism of hepatic insulin resistance in non-alcoholic fatty liver disease. J Biol Chem.

[36] Montonen J, Boeing H, Fritsche A, Schleicher E, Joost H-G, Schulze MB (2013). Consumption of red meat and whole-grain bread in relation to biomarkers of obesity, inflammation, glucose metabolism and oxidative stress. Eur J Nutr.

[37] Fan N, Peng L, Xia Z, Zhang L, Song Z, Wang Y, Peng Y (2019). Triglycerides to high-density lipoprotein cholesterol ratio as a surrogate for nonalcoholic fatty liver disease: a cross-sectional study. Lipids Health Dis.

[38] Avila F, Echeverría G, Pérez D, Martinez C, Strobel P, Castillo O (2015). Serum ferritin is associated with metabolic syndrome and red meat consumption. Oxid Med Cell Longev.

[39] Kowdley KV, Belt P, Wilson LA, Yeh MM, Neuschwander-Tetri BA, Chalasani N (2012). Serum ferritin is an independent predictor of histologic severity and advanced fibrosis in patients with nonalcoholic fatty liver disease. Hepatology.

[40] Pan X, Chen B, Liu W, Li Y, Hu Z, Lin X (2019). Circulating iron levels interaction with central obesity on the risk of nonalcoholic fatty liver disease: a case–control study in Southeast China. Ann Nutr Metab.

[41] Gill V, Kumar V, Singh K, Kumar A, Kim J-J (2019). Advanced glycation end products (AGEs) may be a striking link between modern diet and health. Biomolecules.

[42] Wang Y, Hui T, Zhang YW, Liu B, Wang FL, Li JK (2015). Effects of frying conditions on the formation of heterocyclic amines and trans fatty acids in grass carp (*Ctenopharyngodon idellus*). Food Chem.

[43] Palma-Duran SA, Kontogianni MD, Vlassopoulos A, Zhao S, Margariti A, Georgoulis M (2018). Serum levels of advanced glycation end-products (AGEs) and the decoy soluble receptor for AGEs (sRAGE) can identify non-alcoholic fatty liver disease in age-, sex- and BMI-matched normo-glycemic adults. Metab Clin Exp.

[44] Mazidi M, Katsiki N, Mikhailidis DP, Banach M (2018). Link between plasma trans-fatty acid and fatty liver is moderated by adiposity. Int J Cardiol.

[45] Rietman A, Sluik D, Feskens EJM, Kok FJ, Mensink M (2018). Associations between dietary factors and markers of NAFLD in a general Dutch adult population. Eur J Clin Nutr.

[46] Rouhani MH, Salehi-Abargouei A, Surkan PJ, Azadbakht L (2014). Is there a relationship between red or processed meat intake and obesity? A systematic review and meta-analysis of observational studies. Obes Rev.

[47] Petrović G, Bjelaković G, Benedeto-Stojanov D, Nagorni A, Brzački V, Marković-Živković B (2016). Obesity and metabolic syndrome as risk factors for the development of non-alcoholic fatty liver disease as diagnosed by ultrasound. Vojnosanit Pregl.

[48] Kosmidou M, Milionis H (2017). Diabetes mellitus and non-alcoholic fatty liver disease: the thread of Ariadne. Minerva Endocrinol.

[49] Guo Z, Li M, Han B, Qi X (2018). Association of non-alcoholic fatty liver disease with thyroid function: a systematic review and meta-analysis. Dig Liver Dis.

[50] Liu Y, Wang W, Yu X, Qi X (2018). Thyroid function and risk of non-alcoholic fatty liver disease in euthyroid subjects. Ann Hepatol.

